# Positive Anti-HIV ELISA Results in Pregnancy: Is It Reliable?

**DOI:** 10.1155/2022/1157793

**Published:** 2022-02-16

**Authors:** Emrah Güler, Ayşe Arıkan, Mariam Abobakr, Murat Sayan, Kaya Süer, Tamer Şanlıdağ

**Affiliations:** ^1^Department of Nutrition and Dietetics, Faculty of Health Sciences, Near East University, Nicosia, Cyprus; ^2^DESAM Research Institute, Near East University, Nicosia, Cyprus; ^3^Department of Medical Microbiology and Clinical Microbiology, Faculty of Medicine, Near East University, Nicosia, Cyprus; ^4^Clinical Laboratory, PCR Unit, Faculty of Medicine, Kocaeli University, Kocaeli, Turkey; ^5^Department of Infectious Disease and Clinical Microbiology, Faculty of Medicine, Near East University, Nicosia, Cyprus

## Abstract

Background: Human immunodeficiency virus (HIV) can be transmitted from mothers to their babies during pregnancy, delivery through vaginal fluids or breastfeeding. As false positivity anti-HIV results due to pregnancy could be detected and no relevant study have been reported in Northern Cyprus so far, we aimed to estimate the false anti-HIV positivity rate in pregnant women. Methods: A total of 11.977 women admitted to Near East University Hospital between 2015 and 2018 were involved. The fourth generation anti-HIV-1/2 ELISA test was carried out by chemiluminescence enzyme immunoassay. Positive results were confirmed by real-time polymerase chain reaction (rt-PCR). SPSS (Statistical Package for the Social Sciences) Demo Ver 22 program was used for statistical analysis and significance (p<0.05) was measured by Person Chi-Square and Fisher's Exact tests. Results: Anti-HIV-1/2 ELISA test was positive in 7 (0.3%) of pregnant and 11 (0.1%) of non-pregnant women. HIV RNA was not detected in any pregnant however, was detected in 2 (0.02%) of non pregnant. S/Co titer of pregnant and non pregnant who have positive anti-HIV-test without viral load was x̄=2.68±1.64 (1.34-5.20) and x̄=8.63±7.68 (1.56-20.98) respectively. False positivity was significantly higher in pregnants compared to non-pregnants (p=0.033). Conclusion: False positivity can be encountered during pregnancy therefore, positive anti-HIV-1/2 ELISA results should be confirmed with molecular techniques before initiating antiretroviral treatment.

## 1. Introduction

Acquired immune deficiency syndrome (AIDS) is a potentially life-threatening disease caused by the human immunodeficiency virus (HIV), which attacks immune cells and weakens the immune system that fights infections and diseases [1]. The most common way of getting HIV is through unprotected sexual intercourse, sharing infected needles or other injecting equipment, and transmission from virus-infected mothers to their babies during pregnancy, delivery through vaginal fluids, or breastfeeding [2]. There are two known serotypes of HIV; HIV-1 is common worldwide, and HIV-2 is found mainly in Africa countries. While HIV-2 progresses with lower morbidity and mortality than HIV-1, the AIDS stage takes place many years later, and the rate of transmission from mother to baby is lower than that of HIV-1 (10-40%) [3]. According to 2019 data of the Joint United Nations HIV/AIDS Program/the Joint United Nations Program on HIV/AIDS (UNAIDS), it is reported that 37.9 million people were infected with HIV worldwide at the end of 2018, of those 1.7 million were children under the age of 15 [4]. In Turkey, 1,772 AIDS cases have been reported, and 0.4% of these patients (*n* = 13) acquired AIDS from transmission from mothers to their babies [5]. Although the number of HIV-infected pediatric patients worldwide has been reduced by 50% since 2010, 1,800 new cases of children are reported daily [6]. Northern Cyprus is among the low endemic countries in terms of HIV prevalence. In a recent study, HIV seropositivity was determined as 0.04% in Northern Cyprus [5].

HIV is unlikely to pass on to the child if the mother receives anti-retroviral therapy (ART) treatment during pregnancy and breastfeeding [4]. The World Health Organization (WHO) reported that 54% of HIV-positive children and 82% of HIV-positive pregnant and breastfeeding women received lifetime ART in low- and medium-level countries [3]. These precautions for pediatric HIV infection elimination and their mothers' survival are essential steps in ending the AIDS epidemic [6]. The Centers for Disease Control and Prevention (CDC) recommends that all pregnant women get tested for HIV during each pregnancy. This precaution enables infected women to start prophylaxis and medical care, plan their birth, and prevent transmission through breastfeeding [7]. While the transition rate from mother to baby is 25-30% without taking necessary precautions, this rate can decrease to 2% with initiation of antivirals, predetermination of delivery, and avoiding breastfeeding. Therefore, an anti-HIV-1/2 ELISA screening test is often performed for pregnant women in the prenatal and antenatal period to prevent HIV transmission from mother to baby during pregnancy, delivery, and breastfeeding [8, 9].

**Figure 1:**
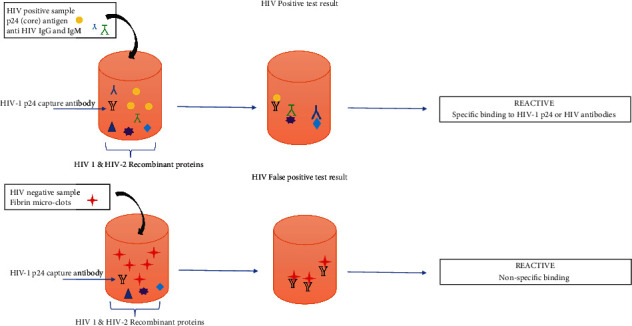
Principles for the fourth-generation HIV antigen/antibody combination assay.

Currently, serological and virological methods can make a laboratory-based diagnosis of HIV infection [10]. Anti-HIV-1/2 screening has been performed since 1985 with the enzyme-linked immunosorbent assay (ELISA) method [11]. The fourth-generation ELISA methods used in diagnosing HIV infection are still preferred today because of their high sensitivity, specificity, and low cost [12]. However, besides factors such as hemolysis, presence of lipemia, anticoagulant, and alloantigens present in the patient's sera, especially during pregnancy, transfusion, transplantation, and autoimmune disease such as human leukocyte antigen (HLA-DR), systemic lupus erythematosus (SLE), rheumatoid arthritis (RA), anti-lymphocyte antibody, and anti-collagen antibody can sometimes generate false positives by cross-reactivity in serological tests (Figure 1) [11, 13, 14]. False positivity is one of the most common concerns, especially in societies with a low incidence of HIV, and pregnant women in these populations are at high risk for false-positive results [15, 16].

As false positivity due to pregnancy could be detected and no relevant study has been reported mainly for pregnant women in Northern Cyprus so far, we aimed to estimate the rate of false positivity in anti-HIV-1/2 ELISA tests conducted in a laboratory in Northern Cyprus and to determine the HIV-1 infection seropositivity in pregnant women.

## 2. Materials and Methods

### 2.1. Patients and Study Design

A total of 11,997 individuals over 18 who applied to Near East University (NEU) Hospital, Nicosia Province, between September 01, 2015, and December 31, 2018, were involved retrospectively in the current study. Among these individuals, 2,495 were pregnant and 9,482 were nonpregnant.

### 2.2. Anti-HIV Screening Test

The anti-HIV-1/2 tests of women were carried out by the chemiluminescence enzyme immunoassay (CEIA) ((HIV 1/2 combo, p24 Ag/Ab) by using Architect i2000 SR, Abbott Laboratories, IL, USA) according to the manufacturer's recommendations. Anti-HIV S/Co (signal-to-cutoff) value was taken as ≥1, and the results above this value were considered positive. Fourth-generation anti-HIV-1/2 ELISA screening test positive samples were centrifuged at high speed, and the tests were repeated using the same assay. The women whose repeated tests were found to be “indeterminant” and “positive” were directed to the Clinical Microbiology and Infectious Diseases specialist.

### 2.3. Molecular Testing

Viral load determination in anti-HIV-1/2 ELISA positive samples was performed by real-time polymerase chain reaction (real-time PCR/RT-PCR). According to the manufacturer's instructions, ribonucleic acid (RNA) was isolated (EZ1 Advanced, Qiagen, Hilden, Germany). For quantitative detection of HIV-1-specific RNA of the isolated samples, *artus* HI Virus-1 RT-PCR on the Rotor-Gene Q instrument was used (*artus* HIV RT-PCR kit, Qiagen, Hilden, Germany).

### 2.4. Statistical Analysis

Statistical analysis of the data obtained was done with the SPSS (Statistical Package for the Social Sciences) Demo ver 22.0 (SPSS Inc., Chicago, IL, USA) program. Pearson's chi-square and Fisher's exact test was used to determine statistical significance, and the matter was evaluated at *p* < 0.05.

## 3. Results

The average age and standard deviations of 2,495 pregnant women (21%) and 9,482 nonpregnant women (79%) included in the study were 29 ± 5 (18-48) and 34 ± 18 (18-97), respectively.

The anti-HIV-1/2 ELISA screening test was found to be positive in 0.3% (7/2,495) of pregnant women; however, no viral load was detected in any of these women by the RT-PCR technique. Therefore, the rate of false anti-HIV-1/2 ELISA positivity in pregnant women was determined to be 0.3%. In nonpregnant women, the rate of anti-HIV-1/2 ELISA positivity was defined as 0.1% (11/9,482). While HIV-1 RNA was not detected in 9 (0.1%) of these women, HIV-1 RNA positivity with viral load values of 3.5 + *E*4 and 1.2 + *E*3 was detected in two (0.02%) of nonpregnant women. False anti-HIV-1/2 ELISA positivity in nonpregnant women was determined as 0.1%.

S/Co titer of women with positive anti-HIV-1/2 ELISA tests without viral load was calculated as $\bar{x}=2.68\pm 1.64$ (1.34-5.20). In nonpregnant women, this value was found to be $\bar{x}=8.63\pm 7.68$ (1.56-20.98). Statistically, false positivity in anti-HIV-1/2 ELISA tests was significantly higher in pregnant women than in nonpregnant women (*p* = 0.033). The age, anti-HIV-1/2 ELISA S/Co values, and HIV-1 RNA results of pregnant and nonpregnant women with positive anti-HIV-1/2 ELISA tests are shown in Table 1. In our study, HIV-1 RNA was not detected in pregnant women. However, HIV-1 RNA was detected in 0.02% of nonpregnant women. There was no statistically significant difference between the detection of HIV-1 RNA and pregnancy status (*p* = 0.627).

## 4. Discussion

This study on pregnant women in Northern Cyprus shows that false positivity can be encountered in the HIV screening serological test routinely performed in our country during pregnancy. The fact that early diagnosis and treatment positively affect HIV infection indicates the necessity of having HIV screening tests for pregnant women in the early periods of pregnancy [17]. In 2018, 62% of adults and 52% of HIV-infected children were reported to receive ART treatment [3]. Due to antiretroviral use between 2000 and 2018, new HIV infections decreased by 37%, and 13.6 million people could survive. UNAIDS aims to identify 90% of HIV-infected people and ensure viral suppression by receiving ART for all HIV-infected individuals in 2030 [4].

Studies show that the number of children infected with HIV under 15 is around 1.7 million worldwide [18, 19]. In Turkey, 199 children have been reported to have HIV infection, and 0.8% (170/21.520) of those have acquired the infection from their mothers [13]. According to the Ministry of Health of the Turkish Republic of Northern Cyprus (TRNC) data, the number of HIV-positive patients was reported to be 11, 20, 7, 7, and 5, respectively, in 2014, 2015, 2016, 2017, and 2018 in Northern Cyprus [20]. However, the TRNC Medical Association notices that as of November 2019, 75 citizens infected with HIV live in Northern Cyprus. Cyprus is an island located in the Eastern Mediterranean region, and this increase in the number of cases may be due to the critical location for human trafficking and immigration [21]. In our study, HIV positivity was not detected in pregnant women, while this rate was determined as 0.02% in nonpregnant women.

The diagnosis of HIV infection is based on serological and virological markers. According to the guidelines for reporting results from the HIV laboratory diagnostic testing algorithm for serum and plasma specimens, the initial diagnosis starts with the HIV-1/HIV-2 antigen/antibody immunoassay. The next step is performing HIV-1/HIV-2 antibody differentiation immunoassay and, finally, the HIV-1 nucleic acid test [22–24]. For Turkey, according to the HIV diagnosis and screening algorithm in the Republic of Turkey Ministry of Health HIV/AIDS Diagnosis and Treatment Guidelines updated in 2019, in adults and children over 18 months, if at least one of the repeat screening tests is positive, a confirmation test should be carried out with an antibody immunoassay test; however, for pregnant women who are in the same condition, it is recommended that the HIV-1 RNA test be performed directly due to a false-positive ELISA result [12, 13]. ELISA tests used for screening are expected to have at least 99% sensitivity. However, these tests can recognize other proteins as HIV-specific antibodies and report false-positive results [13]. Therefore, all samples found to be positive in screening ELISA tests, especially for pregnant women, should be confirmed by nucleic acid testing before initiating ART [12, 17]. Our study did not detect any viral load in pregnant women whose anti-HIV-1/2 ELISA results were positive (*n* = 7). In Northern Cyprus, viral load determination is rapidly done by RT-PCR without confirmation with an antibody test for pregnant women [6]. In addition, prophylaxis is not initiated unless HIV-1 RNA results are reported. This application prevents early diagnosis of possible HIV-1 infection and unnecessary use of ART in mothers and newborns.

All pregnant women with HIV should start to use antiretrovirals during pregnancy for their health and preventing mother-to-child transmissions such as nevirapine, lamivudine, and zidovudine (brand name: Retrovir) that are commonly used as treatment during pregnancy and delivery [8]. The use of HIV medicines, especially zidovudine HIV transmission from HIV-positive pregnant women to babies, ensures a healthy birth. However, inappropriate use of ART may have psychosocial and health problems [25]. Therefore, ensuring the reliability and accuracy of the tests performed can effectively determine the treatment strategy. Similar studies demonstrating false anti-HIV-1/2 ELISA positivity showed that the confirmation tests were negative [6, 26]. Sayan et al. found a statistically significant difference in anti-HIV-1/2 ELISA false positivity in their studies with pregnant and nonpregnant women (*p* = 0.034) [6]. In our research, the false anti-HIV-1/2 ELISA positivity obtained in pregnant women was significantly higher than that in nonpregnant women (*p* = 0.033). These results suggest the necessity of confirmation of positive anti-HIV ELISA results before initiation of ART treatment. Considering the emotional and psychological changes experienced during pregnancy by hormonal changes, it should be regarded that false-positive results in screening tests may hurt pregnant women [27]. Pregnant women may want to give up their baby, or mothers who give birth can avoid breastfeeding their babies.

To point out this study's limitation, we repeated the anti-HIV-1/2 ELISA screening test positive samples using the same assay after high-speed centrifugation to eliminate cross-reaction with fibrin microclots. However, we did not report these values because the results obtained in the second repetition were similar to the first results. Therefore, we could not provide repeated anti-HIV 1/E ELISA values.

## 5. Conclusion

In conclusion, as false positivity can be encountered during pregnancy, this study suggests that positive anti-HIV-1/2 ELISA results should be confirmed with molecular techniques before ART is initiated. In addition, we also believe that HIV-2 should also be considered alongside HIV-1 due to human trafficking and migration from all over the world to Northern Cyprus. Patients with suspected HIV-1 infection should also be investigated for HIV-2 infection in Northern Cyprus. In the future, immunological tests that differentiate HIV-1/2, which can detect HIV-1 and HIV-2 infections, and then performing nucleic acid tests for both infections will be the most accurate approach in determining HIV infections in our country.

## Figures and Tables

**Table 1:** Age, anti-HIV S/Co values, and HIV-1 RNA test results of pregnant and nonpregnant women with positive anti-HIV ELISA tests.

Study patient	Age	Anti-HIV screening	HIV-1 RNA
		Architect HIV 1/2 Combo p24 Ag/Ab, S/Co	Artus HIV-1 RT-PCR
Pregnant women			
1.	25	5.20	Not detected
2.	22	4.86	Not detected
3.	27	2.14	Not detected
4.	31	1.93	Not detected
5.	29	1.87	Not detected
6.	30	1.39	Not detected
7.	48	1.34	Not detected
Average	30	2.68	
Nonpregnant women			
1.	28	20.98	Not detected
2.	22	18.70	Not detected
3.	27	13.81	Not detected
4.	26	10.77	Not detected
5.	67	5.38	Not detected
6.	22	2.74	Not detected
7.	40	1.98	Not detected
8.	25	1.78	Not detected
9.	25	1.56	Not detected
Average	31	8.63	
10.	44	157.87	3.5 + *E*4
11.	27	52.10	1.2 + *E*3
Average	36	104.99	

Abbreviations: HIV: human immunodeficiency virus; S/Co: signal-to-cutoff; gp24: glycoprotein 24; RT-PCR: reverse transcriptase-polymerase chain reaction.

## Data Availability

All data of the study can be obtained from the corresponding author upon request.

## Supplementary Materials

Supplementary Materials Full datasets and other materials about this study could be obtained from the corresponding author upon reasonable request.
