# Supplementary figures and images for: Positive Anti-HIV ELISA Results in Pregnancy: Is It Reliable?
Source: Infect Dis Obstet Gynecol. 2022 Feb 16;2022:1157793. doi: 10.1155/2022/1157793 (PMC8865967; doi:10.1155/2022/1157793)

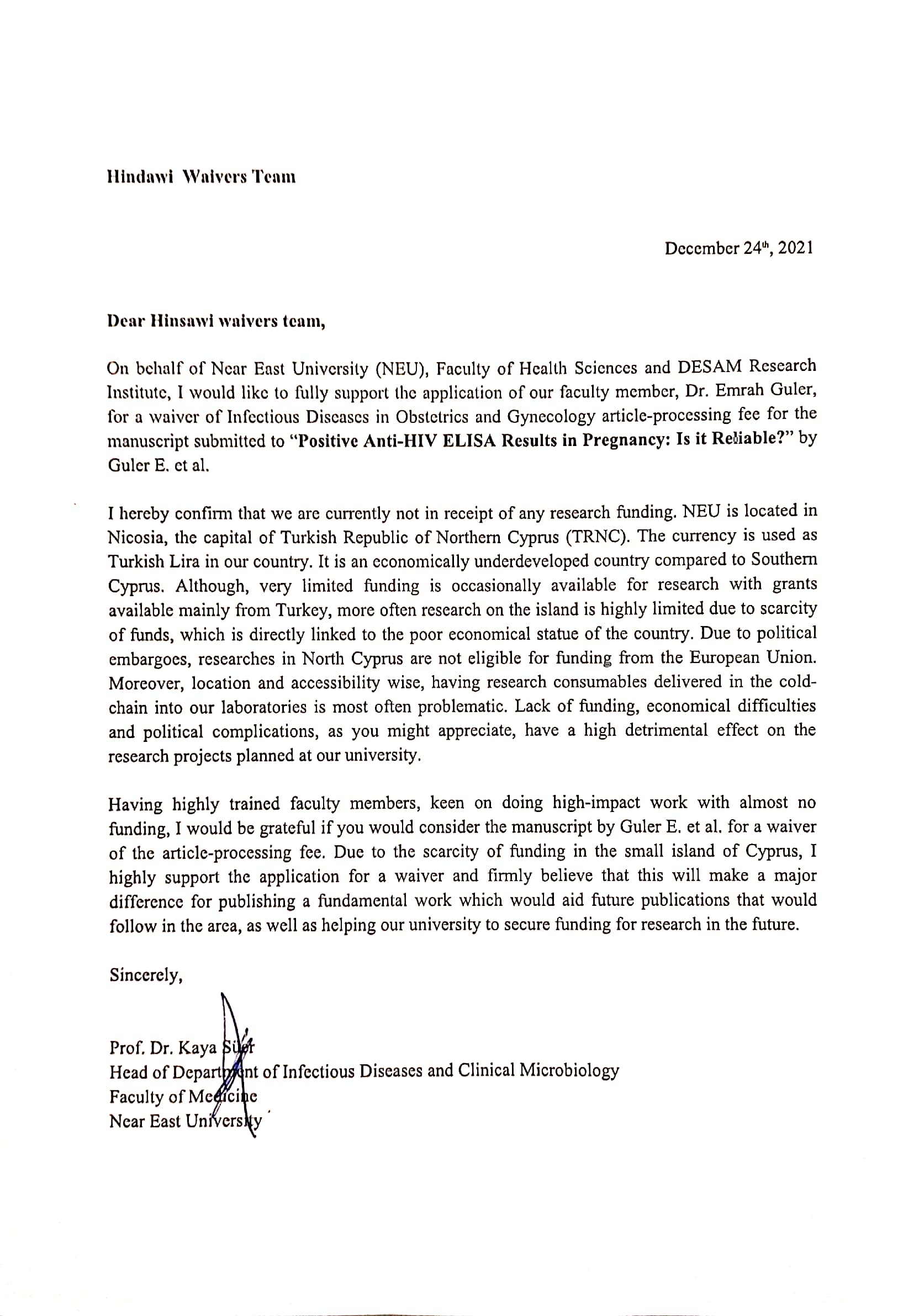

Supplement: Supplementary Materials — Full datasets and other materials about this study could be obtained from the corresponding author upon reasonable request. [file 1157793.f1.jpg]
